# A rare case of glial fibrillary acidic protein astrocytopathy that resolved spontaneously within a self-limited course^☆^

**DOI:** 10.1016/j.heliyon.2023.e20912

**Published:** 2023-10-12

**Authors:** Mihiro Kaga, Takeshi Ueda, Satoshi Yoshikawa

**Affiliations:** a Emergency and General Internal Medicine, Rakuwakai Marutamachi Hospital, Kyoto, Japan; b Department of Clinical Biostatistics, Graduate School of Medical and Dental Sciences, Tokyo Medical and Dental University, Tokyo, Japan

**Keywords:** Autoimmune meningoencephalomyelitis, cerebrospinal fluid, glial fibrillary acidic protein

## Abstract

Glial fibrillary acidic protein astrocytopathy is a form of autoimmune meningoencephalomyelitis. The presence of antibodies in spinal fluid against glial fibrillary acidic protein is necessary to diagnose the disease. There is no standard treatment and few cases of glial fibrillary acidic protein astrocytopathy have been reported. A 31-year-old healthy Japanese man presented to our emergency department with a 7-day history of fever and headache. He was in good general condition, without abnormalities on physical examination, and a general hematological examination revealed hyponatremia (130 mEq/L). Five days later, he was followed up and new subjective symptoms were noted: tremor in the right hand, constipation, sweating, and lightheadedness. Cerebrospinal fluid examination revealed a cell count of 57/μL (96 % mononuclear cells, 4 % multinuclear cells), elevated protein level (103 mg/dL), elevated adenosine deaminase level (15.0 U/L), negative polymerase chain reaction test results for herpes simplex virus and *Mycobacterium tuberculosis*, negative cerebrospinal fluid culture, and negative cerebrospinal fluid anti-acid bacteria culture, indicating aseptic meningitis. T1-weighted contrast-enhanced magnetic resonance imaging of the head showed a linear contrast effect perpendicular to the lateral ventricular wall and along the perivascular vessels spreading radially. Based on the presence of hyponatremia, history of movement disorder and autonomic symptoms, high adenosine deaminase level in cerebrospinal fluid, and findings on contrast-enhanced magnetic resonance imaging of the head, we suspected glial fibrillary acidic protein astrocytopathy and assessed anti-glial fibrillary acidic proteinαantibody in cerebrospinal fluid, which was positive, and diagnosed glial fibrillary acidic protein astrocytopathy. After careful follow-up with symptomatic treatment without immunosuppressive therapy, the fever, headache, tremor, and autonomic symptoms were improved over time. Contrast-enhanced magnetic resonance imaging of the head and findings of cerebrospinal fluid also showed improvement. glial fibrillary acidic protein astrocytopathy should be a differential diagnosis in patients with aseptic meningitis with movement disorders or autonomic symptoms and elevated cerebrospinal fluid adenosine deaminase. Careful follow-up without immunosuppressive treatment should be considered for patients with minimal neurologic symptoms as glial fibrillary acidic protein astrocytopathy may have a self-limiting course and resolve.

## Highlights

• Glial fibrillary acidic protein astrocytopathy (GFAP-A) is an autoimmune disease.
• Movement disorder and autonomic symptoms are common in GFAP-A.
• GFAP-A causes elevated adenosine deaminase levels and hypoglycorrhachia.
• These symptoms and findings can be misdiagnosed, leading to unnecessary treatments.
• Consider follow-up without immunosuppressive therapy for GFAP-A with mild symptoms.

## 1 Introduction

Glial fibrillary acidic protein astrocytopathy (GFAP-A) is a form of autoimmune meningoencephalomyelitis first reported in 2016 [1]. Glial fibrillary acidic protein (GFAP) serves a number of important functions in the central nervous system and is important for maintaining the mechanical strength of astrocytes and supporting adjacent neurons [2]. The presence of antibodies in the cerebrospinal fluid (CSF) to GFAP, the major cytoskeletal structural protein of astrocytes, is necessary to diagnose this disease [3]. Immunosuppressive therapy with corticosteroids is commonly used, although no standard treatment has been established [4]. Few cases of GFAP-A have been reported to date, and further case reports are needed [5]. In this report, we describe the first case report of GFAP-A that resolved spontaneously after a self-limited course.

## 2 Case presentation

**Fig. 1:**
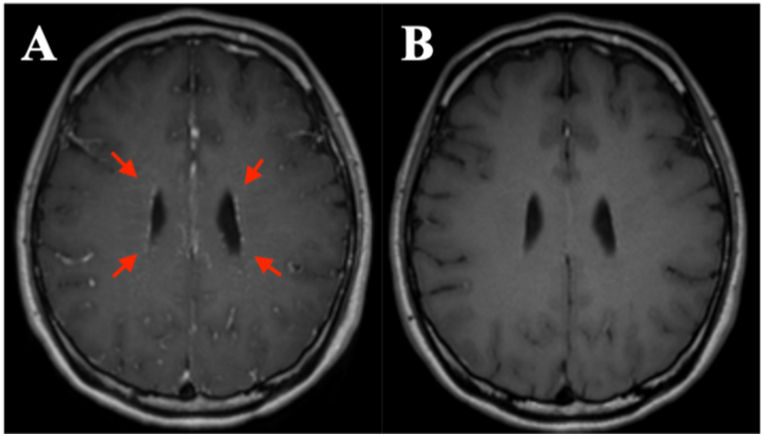
MRI before and after treatment A) Pre-treatment images. Contrast-enhanced T_1_-weighted MRI scan of the head shows a linear contrast effect perpendicular to the lateral ventricular wall and along the perivascular vessels spreading radially (red arrows). Anti-GFAPα antibody was detected in the cerebrospinal fluid of the patient and a diagnosis of GFAP-A was made. Half of the patients with GFAP-A show this sign [4]. B) Post-treatment images. The patient was treated symptomatically. Loxoprofen 60 mg or acetaminophen 400 mg was prescribed as needed (pro re nata), depending on the pain. A linear contrast effect that was present before treatment has disappeared. MRI, magnetic resonance imaging GFAP-A, glial fibrillary acidic protein astrocytopathy.

**Fig. 2:**
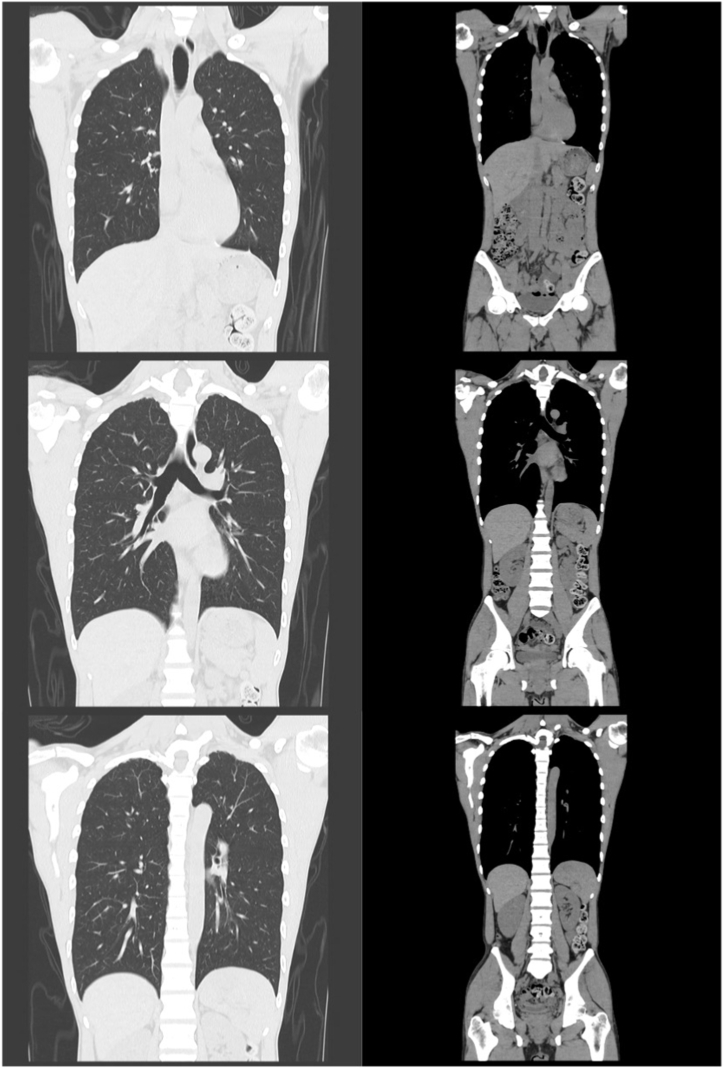
CT before treatment Pre-treatment images. Thoracoabdominal CT scan showed no abnormal findings. There were reports of malignant neoplasms within 3 years of GFAP-A onset, but none were identified in this patient at this time [1,6]. CT, computed tomography GFAP-A, glial fibrillary acidic protein astrocytopathy.

A healthy 31-year-old Japanese man, who was a non-smoker without any history of alcohol consumption, presented to our emergency department with a 7-day history of fever and headache. His vital signs were normal except for a fever of 38.3 °C. His physical examination revealed no abnormalities. General hematological examination revealed hyponatremia (130 mEq/L). Severe acute respiratory syndrome coronavirus 2 nucleic acid amplification test result was negative. He was followed up 5 days later at the outpatient clinic for a suspected viral infection. A few days before his second visit to the department, he reported the presence of a tremor in his right hand, constipation, sweating, and lightheadedness. When he visited our hospital, his consciousness was clear and he was cooperative. Neurological examination showed no special findings except for the presence of postural tremors in both upper limbs. CSF examination revealed a cell count of 57 cells/μL (96 % mononuclear cells, 4 % multinuclear cells), elevated protein level (103 mg/dL), elevated adenosine deaminase (ADA) level (15.0 U/L), and hypoglycorrhachia (41 mg/dL). Additionally, polymerase chain reaction test results for herpes simplex virus and *Mycobacterium tuberculosis* were negative. CSF culture and CSF acid-fast bacillus culture results were also negative. T1-weighted contrast-enhanced magnetic resonance imaging (MRI) of the head showed a linear contrast effect perpendicular to the lateral ventricular wall and along the perivascular vessels spreading radially (Fig. 1A). Thoracoabdominal computed tomography (CT) scan showed no abnormal findings (Fig. 2). Additional assessments subsequently revealed no abnormalities in thyroid-stimulating hormone (0.945 μIU/mL), free thyroxine (1.51 ng/dL), serum complement titer (45.0 CH50/mL), C3 (102 mg/dL), C4 (29 mg/dL), and angiotensin-converting enzyme (8.1 U/L) levels. The results of antinuclear antibody, anti-cyclic citrullinated peptides antibodies, anti-double-stranded deoxyribonucleic acid (DNA) IgG antibody, anti-Sjogren's syndrome A/Ro antibody, anti‐DNA antibody, β-D glucan, and cryptococcus antigen tests were negative. Furthermore, hepatitis B surface antigen, hepatitis C virus antibody, serological test for syphilis, Treponema pallidum antibody, and human immunodeficiency virus antigen/antibody combination tests were negative. In addition, anti-aquaporin-4, anti-myelin-oligodendrocyte glycoprotein, paraneoplastic syndrome-related antibodies, and anti-glutamic acid decarboxylase antibodies were absent, and the soluble interleukin-2 receptor level was normal (208 U/mL).

Based on the above, the patient was treated symptomatically for suspected aseptic meningitis and followed up. Loxoprofen 60 mg or acetaminophen 400 mg was prescribed as needed (pro re nata), depending on the pain. Loxoprofen was indicated to be taken up to three times a day at a maximum of one 60 mg tablet spaced at least 6 hours apart, and acetaminophen was indicated to be taken up to four times a day at a maximum of two 200 mg tablets spaced at least 6 hours apart, at the patient's discretion. At 41 days from onset, CSF cell count and ADA had improved to 22/μL and 3.1 U/L, respectively. A sample of CSF was tested for anti-GFAPα antibody only once, using a sample of CSF on day 41 of onset. On day 55 of onset, anti-GFAPα antibody was detected in that CSF, and a diagnosis of GFAP-A was made. The fever, headache, tremor, autonomic symptoms, and CSF findings improved (a cell count of 22 cells/μL, protein level of 52 mg/dL, and ADA level of 3.1 U/L on day 41 of onset) over a period of approximately 40 days but slight fatigue remained. In addition, contrast-enhanced MRI findings of the head had also improved at 118 days after symptom onset (Fig. 1B). Currently, over 8 months have passed from disease onset to the last follow-up date, and no recurrence of GFAP-A has been observed. No adverse events occurred during the course of the treatment.

## 3 Discussion

To our knowledge, this is the first case report of spontaneous resolution of GFAP-A after a self-limited course. Treatment without immunosuppressive therapy with corticosteroids, as in this case, avoided the adverse events that can occur with immunosuppressive therapy. Long-term use of corticosteroids may be associated with serious sequelae (i.e., osteoporosis; aseptic joint necrosis; adrenal insufficiency; gastrointestinal, hepatic, and ophthalmologic effects; hyperlipidemia; and growth suppression) and possible birth defects [7]. The spontaneous resolution of GFAP-A in a self-limited course suggests that the anti-GFAPα antibody itself is not pathogenic and that other factors may be pathogenic. There have been case reports of GFAP-A after viral infection, and GFAP-A may be a transient immune response after infection [5]. There are some hypotheses that activation of GFAP-specific CD8-positive T lymphocytes may cause the inflammatory pathology [1,8]. The pathogenic mechanism of anti-GFAPα antibodies remains to be fully elucidated.

Tremor, a form of movement disorder, and autonomic symptoms are common in GFAP-A [3,4]. Self-limited neurological symptoms have also been reported in children [9]. Laboratory findings in GFAP-A patients are characterized by hyponatremia, elevated levels of ADA in CSF, hypoglycorrhachia, and elevated CSF cell counts lasting for months [4,10]. Half of the patients with GFAP-A show a radial, perivascular linear contrast effect perpendicular to the lateral ventricular wall on contrast-enhanced MRI of the head [4]. These findings are consistent with those of our case. This MRI finding is not specific to GFAP-A, as it can be seen in diseases other than GFAP-A [4]. In other words, the diagnosis of GFAP-A cannot be made based on this MRI finding alone but requires testing for anti-GFAPα antibodies in CSF [3,4]. Tuberculous meningitis is a well-known disease with high CSF ADA levels and hypoglycorrhachia, but these findings have also been reported in autoimmune-related central nervous system diseases, such as multiple sclerosis [10,11]. ADA plays a central role in the differentiation and maturation of T lymphocytes, which are critical for cell-mediated immune responses [12]. This fact is consistent with previous reports suggesting that GFAP-A is associated with CD8-positive T lymphocytes. It is possible that GFAP-A has been misdiagnosed as tuberculous meningitis based on elevated CSF ADA levels and hypoglycorrhachia or in cases diagnosed as aseptic meningitis that have been improved [10]. This causes unnecessary administration of multidrug antituberculosis regimens and side effects. The present patient had a relatively subacute course with persistent fever and headache and mild neurological findings. As the patient was in good general condition and the CSF cell count was elevated with mononuclear cell predominance, we considered the patient to have aseptic meningitis and decided to follow him up for further evaluation. The patient was later found to be positive for anti-GFAPα antibody in CSF, and a diagnosis of GFAP-A was made.

GFAP-A is often reported to occur with moderate or severe neurological impairment of modified Rankin Scale 3 or higher, but this case showed mild neurological impairment [13]. In a previous report, the median modified Rankin Scale after treatment was 1, and all known treatment results occurred after immunosuppressive therapy [6]. The present case did not receive immunosuppressive treatment and had a similar outcome as that in the report. It has been reported that approximately 20–50 % of patients with GFAP-A will relapse [4] and there was also a report of a malignant neoplasm within 3 years after the onset of the disease, indicating that long-term follow-up and evaluation with 18- fluorodeoxyglucose-positron emission tomography should also be considered [1,6]. Recurrent cases may be treated with mycophenolate mofetil, azathioprine, rituximab, and cyclophosphamide [4].

## 4 Conclusion

GFAP-A should be a differential diagnosis in patients with aseptic meningitis with movement disorders or autonomic symptoms and elevated CSF ADA. Careful follow-up without immunosuppressive treatment should be considered for patients with minimal neurologic symptoms as GFAP-A may have a self-limiting course and resolve.

## Financial support

The authors did not receive any financial support.

## Ethical approval

Written informed consent was obtained from the patient for publication of this report. Ethics approval for this study was provided by the ethics committee of Rakuwakai Marutamachi Hospital (ethical approval number: 02-22-00027), and the study was conducted in accordance with the guidelines of the Declaration of Helsinki.

## Data availability

No data was used for the research described in the article.

## CRediT authorship contribution statement

**Mihiro Kaga:** Conceptualization, Writing – original draft. **Takeshi Ueda:** Supervision. **Satoshi Yoshikawa:** Supervision.

## Declaration of competing interest

The authors declare that they have no known competing financial interests or personal relationships that could have appeared to influence the work reported in this paper.

## Abbreviations

GFAP-A: glial fibrillary acidic protein astrocytopathy
GFAP: glial fibrillary acidic protein
CSF: cerebrospinal fluid
ADA: adenosine deaminase
MRI: magnetic resonance imaging
CT: computed tomography
DNA: deoxyribonucleic acid
